# Ophthalmic Neuro-Myology

**Published:** 1906-06

**Authors:** A. G. Hobbs

**Affiliations:** Atlanta, Ga.


### BOOK REVIEWS.

---

OPHTHALMIC NEURO-MYOLOGY. A study of ocular muscles from the brain side of the question. By G. C. S. Savage, M.D., author of "New Truths and Ophthalmology," "Ophthalmic Myology," and Professor of Ophthalmology in Vanderbilt University, Nashville, Tenn.

It is interesting to have even one brain center definitely located, but in this little book we are given all the separate and co-ordinate centers of the ocular muscles, which we hope will help to explain some of the heretofore unexplicable aberrations of vision. We also have the anterior pole of the visual axis decentered, usually inward from the center of the cornea, which suggests a stronger or weaker glass, as the case may be, when adjusting lens binocularly.

A theory is none the less likely to be true because it is beautiful, nor should it be caviled at, or doubted because of its intricacy, by those who do not give themselves the trouble to study it. The author says in his preface that he was tempted to call his book "Eye Muscle Study Made Easy." Could he not so call his next edition when he has enlarged and amplified it, by including the natural corollaries of his proposition and also the collateral features? That would make the book still more valuable to the refractionist and easier to be grasped by those who are by nature or by acquirement more mechanically than mathematically inclined —more inclined to resort to surgery than to the lens—as a means to the end.

Dr. Savage will no doubt be questioned on some of his mathematical deductions based on brain centers reserved for pathological muscular incentive, but the critic should first be well prepared to substitute some better theory. In the meantime the little book is well worth the close study of every refractionist.

A. G. HOBBS, M.D.

Atlanta, Ga.

